# Temporal evolution of antimicrobial resistance among *Neisseria gonorrhoeae* clinical isolates in the most populated South American Metropolitan Region

**DOI:** 10.1590/0074-02760190079

**Published:** 2019-08-12

**Authors:** Rafael Affini Martins, Dandara Cassu-Corsi, Carolina Silva Nodari, Rodrigo Cayô, Larissa Natsumeda, Ana Paula Streling, André Mario Doi, Roberto José Carvalho da Silva, Roberta Alessandra Lima Bocalon, Ana Cristina Gales, Antonio Carlos Campos Pignatari

**Affiliations:** 1 Universidade Federal de São Paulo, Escola Paulista de Medicina, Departamento de Medicina, Laboratório ALERTA, São Paulo, SP, Brasil; 2 Universidade Federal de São Paulo, Instituto de Ciências Ambientais, Químicas e Farmacêuticas, Departamento de Ciências Biológicas, Laboratório de Bacteriologia e Imunologia, Diadema, SP, Brasil; 3 Universidade Federal de São Paulo, Escola Paulista de Medicina, Departamento de Medicina, Laboratório Especial de Microbiologia Clínica, São Paulo, SP, Brasil; 4 Centro de Referência e Tratamento de Doenças Sexualmente Transmissíveis, São Paulo, SP, Brasil

**Keywords:** Neisseria gonorrhoeae, antimicrobial resistance temporal evolution, azithromycin, ciprofloxacin-non-susceptible

## Abstract

A total of 124 *Neisseria gonorrhoeae* isolates recovered during a 12-year period (2003-2015) from outpatients assisted at Centro de Referência e Treinamento DST/AIDS-CRT of São Paulo city, Brazil, were analysed. The following resistance rates were observed: penicillin-59.6%, ciprofloxacin-15.3%, and azithromycin-6.7%. Although reduced susceptibility to these drugs was observed since 2003, no ceftriaxone-resistant isolates were detected. Ciprofloxacin- and azithromycin non-susceptible isolates were grouped in 11 clusters. Mutations were detected in GyrA and ParC of isolates 124 and 260, and a C_2611_T substitution on 23S rRNA alleles was also observed in isolate 260. Both isolates belonged to ST1901/ST6210 (MSLT/NG-MAST schemes).

*Neisseria gonorrhoeae* is a fastidious Gram-negative human pathogen, which commonly infects the urogenital, rectal, and/or nasopharyngeal mucosa, causing asymptomatic and symptomatic sexually transmitted infections (STI).^1^ ^,^ ^2^ According to the World Health Organization’s (WHO) “Global action plan to control the spread and impact of antimicrobial resistance in *N. gonorrhoeae*”, 78 million new cases of gonorrhea occurred among adults and adolescents aged 15-49 years worldwide,^1^ ^,^ ^3^ with high prevalence in Western Pacific, South-East Asia, and Africa regions.^2^ ^,^ ^3^ The absence of effective vaccines and the inadequate treatment contributed to the re-emergence of this disease and the emergence of multidrug resistant (MDR) strains.^2^

By the end of the 1970s, β-lactamase-producing *N. gonorrhoeae* strains presenting high-level resistance to penicillin spread worldwide.^2^ Currently, plasmid- (*bla*_TEM-1_ or *bla*_TEM-135_ genes) and/or chromosome- (PBP2/PBP1 mutations or overexpression of efflux pump MtrCDE) mediated resistance to penicillin are commonly found among gonococcal strains.^1^ ^,^ ^2^ Therefore, single 250 mg doses of ciprofloxacin had been initially prescribed to treat penicillin-resistant *N. gonorrhoeae* strains, with a further increase in ciprofloxacin dosage to 500 mg.^2^ However, quinolone resistance developed and spread quickly worldwide.^2^ ^,^ ^4^ Combined therapy of intramuscular ceftriaxone (250 mg) and azithromycin (1 g) has been recommended for the treatment of uncomplicated MDR gonococcal infection, following the “WHO Guidelines for the Treatment of *N. gonorrhoeae*” (https://www.who.int/). However, azithromycin-resistant *N. gonorrhoeae* strains have also been described worldwide, combined to an increasing number of studies reporting the emergence of therapeutic failures with cefixime and ceftriaxone, mainly in Asia, Europe, and recently in North America.^1^ ^,^ ^2^ ^,^ ^4^ In Brazil, the antimicrobial susceptibility profile of *N. gonorrhoeae* recovered from distinct geographic regions was recently published.^4^ ^,^ ^5^ ^,^ ^6^ Nevertheless, national studies that correlate the clonal relationship of non-susceptible *N. gonorrhoeae* isolates with the spread of mechanisms of antimicrobial resistance in such pathogen are still lacking. Herein, we described the molecular epidemiology of ciprofloxacin- and azithromycin-non-susceptible *N. gonorrhoeae* clinical isolates recovered in the STI reference center of the São Paulo Metropolitan Region, Brazil, between 2003 and 2015.

A total of 124 *N. gonorrhoeae* clinical isolates belonging to a broader collection stored at the Centro de Referência e Tratamento de Doenças Sexualmente Transmissíveis/AIDS (CRT-DST/AIDS) located in the city of São Paulo, Brazil, was included in the study. The clinical isolates were recovered from urogenital and nasopharyngeal swabs of male (n = 117/124; 94.35%) and female (n = 2/124; 1.62%) outpatients between 2003 and 2015. The São Paulo Metropolitan Region is the most populated area in South America with an estimated population of 21.2 million inhabitants. Isolates recovery and previous species identification were performed following CRT-DST/AIDS standardised protocols applied by the time of clinical specimens’ collection. Clinical isolates were stored at -80ºC until further characterisation. All *N. gonorrhoeae* isolates were subcultured on chocolate agar plates (bioMérieux, Marcy l’Etoile, France), and incubated at 37ºC with 5% CO_2_ for 20-24h. Species identification was confirmed by MALDI-TOF MS using the Microflex LT spectrometer and the Biotyper 3.3 software (Bruker Daltonics, Massachusetts, USA), according to manufacturer’s recommendations.

Minimal inhibitory concentrations (MICs) of penicillin, ceftriaxone, ciprofloxacin, and azithromycin (Sigma-Aldrich, St. Louis, USA) were determined by agar dilution method using Oxoid™ GC agar base (Thermo Fisher Scientific, Basindstoke, UK) supplemented with 1% hemoglobin (Thermo Fisher Scientific, Basindstoke, UK), and Oxoid™ Vitox enrichment (Thermo Fisher Scientific, Basindstoke, UK). The results were interpreted according to the European Committee on Antimicrobial Susceptibility Testing breakpoints Version 5.0 (EUCAST, 2015; available at http://www.eucast.org). *N. gonorrhoeae* ATCC 49226™ strain (American Type Culture Collection) was used as a quality control strain.

Among the *N. gonorrhoeae* isolates, 59.6% (74/124), 15.3% (19/124), and 6.7% (8/119) were resistant to penicillin, ciprofloxacin, and azithromycin, respectively [Figure, Supplementary data (Table)]. An increase in the resistance to ciprofloxacin was observed between 2003 and 2015 [Supplementary data (Fig. 1)]. Similar data was obtained by a previous study conducted in Rio de Janeiro (16.5%), considered the second most populated metropolitan region of Brazil, between 2006 and 2010.^4^ Due to socio-geographic differences among the Brazilian regions, the ciprofloxacin resistance rates change considerably according to the geographic region.^5^ ^,^ ^6^ ^,^ ^7^ Ceftriaxone has been used as the last empirical monotherapy to treat gonococcal infections, and the emergence of resistance to this β-lactam has been considered a worldwide public health concern by WHO (https://www.who.int/). Fortunately, no ceftriaxone non-susceptible isolate was detected in this study (MICs, ≤ 0.001 - 0.06 mg/L; Figure), corroborating with previous findings.^5^ ^,^ ^6^ ^,^ ^7^

**Figure:**
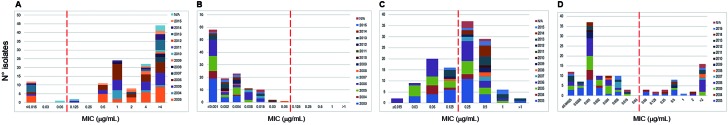
Minimal inhibitory concentration (MIC) distributions for penicillin (A), ceftriaxone (B), azithromycin(C), and ciprofloxacin (D) of 124 *Neisseria gonorrhoeae* isolated from the metropolitan region of São Paulo, Brazil. The red dashed line represents the EUCAST susceptibility breakpoint. NA: data not available.

The genetic relationship of ciprofloxacin- and azithromycin-non-susceptible *N. gonorrhoeae* isolates was determined by pulsed-field gel electrophoresis (PFGE) using CHEF DR II system (Bio-Rad, California, USA) and the SpeI restriction enzyme (New England BioLabs, MA, USA). The restriction patterns were identified and interpreted by dendrogram and cluster analysis performed using algorithms available within the BioNumerics software package v.6.0 (Applied Maths, Sint-Martens-Latem, Belgium). Percentage of similarity between different fingerprints was scored by the Dice coefficient. The unweighted pair group method with arithmetic means (UPGMA) was used to obtain the dendrogram accepting a 1.5% tolerance limit. Although Uehara and colleagues reported a single clone of ciprofloxacin- and azithromycin-resistant *N. gonorrhoeae* isolates spread in the city of Rio de Janeiro,^5^ we observed at least two major ciprofloxacin- and azithromycin non-susceptible *N. gonorrhoeae* clones during the 12-year period of study [Supplementary data (Fig. 2)]. According to PFGE analysis, such isolates were grouped in eleven distinct clusters. The clone A was the most frequent (n = 14), being recovered between 2005 and 2013, followed by clone I (n = 12; 2003 to 2014). On the other hand, clones D and L were represented by a single isolate each, recovered in 2003 and 2005, respectively. The genetic cluster E (n = 5) was mostly composed by ciprofloxacin- and azithromycin non-susceptible *N. gonorrhoeae* isolates, which were recovered in distinct periods [Supplementary data (Fig. 2)].

A single representative of each ciprofloxacin and/or azithromycin non-susceptible genetic cluster was selected for evaluation of mutations in the Quinolone Resistance Determinant Region (QRDR) of *gyrA* and *parC* genes and mutations in the ribosomal 23S rRNA encoding gene, respectively. The genomic DNA of each strain was extracted and purified using Chelex^®^ (Sigma Aldrich, St. Louis, USA) according to manufacturer instructions. The regions of interest were amplified using specific primers previously described,^8^ ^,^ ^9^ and sequencing reactions were prepared using the BigDye Terminator Cycle Sequencing Kit (Applied Biosystems, Foster City, USA) and performed with the ABI 3500 Genetic Analyzer (Applied Biosystems, Perkin Elmer, USA). The nucleotide sequences and the derived protein sequences obtained were compared to the ones of *N. gonorrhoeae* FA 1090 reference strain using the LaserGene Software Package (DNASTAR, Madison, USA). A total of seven representative isolates of each genetic cluster comprising ciprofloxacin-non-susceptible *N. gonorrhoeae* isolates was selected for further analysis. Only two of them, namely 124 and 260, which belonged to clones A and E, respectively, presented point mutations in both GyrA (Ser_91_Phe and Asp_95_Gly) and ParC (Ser_87_Arg), corroborating with a previous Brazilian study that also reported mutations in both genes among ciprofloxacin-resistant *N. gonorrhoeae*.^5^ Interestingly, despite their reduced susceptibility to fluoroquinolones (MICs ranging from 0.25 to > 2 mg/L), most ciprofloxacin-non-susceptible *N. gonorrhoeae* isolates did not present any mutations in *gyrA* and *parC*. These results could suggest that other mechanisms, such as the overexpression of the NorM efflux pump^2^, might be involved in such resistance phenotype. Additionally, representative azithromycin-non-susceptible *N. gonorrhoeae* isolates were selected for further characterisation (n = 9). Among them, only a single isolate (260) showed a C2611T mutation in the four alleles of the 23S rRNA encoding gene. This substitution was previously reported in two macrolide-resistant *N. gonorrhoeae* strains (MICs, 64 and 4 µg/mL for erythromycin and azithromycin, respectively) recovered in Canada during the 1990s and usually confers low-level macrolide resistance.^9^ As observed with the ciprofloxacin non-susceptible isolates, additional resistance mechanisms might be present.

The isolates that presented mutations at *gyrA, parC*, and/or at the 23S rRNA encoding gene were further typed following both *N. gonorrhoeae* Multiantigen Sequencing Typing (NG-MAST) (http://www.stdgen.lanl.gov/stdgen/bacteria/ngon) and Multi Locus Sequence Typing (MLST) schemes (https://pubmlst.org/neisseria/). Both ciprofloxacin and azithromycin-non-susceptible isolates that presented mutations in the investigated genes (124 e 260) belonged to ST1901 following MLST scheme. Interestingly, such isolates were recovered in 2013, contrasting with the report of Barros dos Santos and colleagues, which observed the emergence of azithromycin-resistant *N. gonorrhoeae* belonging to ST1901 only after 2016.^10^ The NG-MAST typing also revealed the same ST for both isolates (ST6210). Such sequence type has been previously deposited at the online database, but, to the best of our knowledge, it has not been published elsewhere.

In conclusion, the present study showed an alarming reduction in the susceptibility rates to penicillin, ciprofloxacin, and azithromycin in *N. gonorrhoeae* in the Metropolitan Region of São Paulo, Brazil, since 2003. Recently, Bazzo and colleagues have reported a 37.1% resistance rate to benzylpenicillin among 550 *N. gonorrhoeae* isolates recovered from adult male patients in five distinct Brazilian cities between October 2015 and December 2016.^7^ The same study also reported high resistance rates to ciprofloxacin (55.6%),^7^ corroborating with data recovered in North America, Europe, and Asia.^1^ We also observed the spread of distinct ciprofloxacin- and/or azithromycin-non-susceptible *N. gonorrhoeae* clones during a 12-year period, demonstrating the high capacity of this pathogen to cause infection in humans. Although resistance to ceftriaxone was not observed, surveillance efforts should be made for the early detection of resistant strains, in order to avoid its dissemination in this geographic region and across the country.

*Ethics* - Ethical approval for this study was obtained from Research Ethics Committee from Federal University of São Paulo ― UNIFESP/Hospital São Paulo (Process number: 1.346.587).

## References

[1] Unemo M, Del Rio C, Shafer WM (2016). Antimicrobial Resistance Expressed by Neisseria gonorrhoeae A
major global public health problem in the 21st century. Microbiol Spectr.

[2] Unemo M, Shafer WM (2014). Antimicrobial resistance in Neisseria gonorrhoeae in the 21st
century past, evolution, and future. Clin Microbiol Rev.

[3] Newman L, Rowley J, Vander Hoorn S, Wijessoriya NS, Unemo M, Low N (2015). Global estimates of the prevalence and incidence of four curable
sexually transmitted infections in 2012 based on systematic review and
global reporting. PLoS One.

[4] Starnino S, GASP-LAC Working Group.Galarza P.Carvallo ME.Benzaken AS.Ballesteros
AM (2012). Retrospective analysis of antimicrobial susceptibility trends
(2000-2009) in Neisseria gonorrhoeae isolates from countries in Latin
America and the Caribbean shows evolving resistance to ciprofloxacin,
azithromycin and decreased susceptibility to ceftriaxone. Sex Transm Dis.

[5] Uehara AA, Amorin EL, Ferreira MF, Andrade CF, Clementino MB, de Filippis I (2011). Molecular characterization of quinolone-resistant Neisseria
gonorrhoeae isolates from Brazil. J Clin Microbiol.

[6] Ferreira WA, Ferreira CM, Naveca FG, Vasconcelos WS, Gomes IS (2013). Molecular epidemiology of ß-lactamase-producing Neisseria
gonorrhoeae strains in Manaus, AM, Brazil. Sex Transm Dis.

[7] Bazzo ML, Golfetto L, Gaspar PC, Pires AF, Ramos MC, Franchini M (2018). First nationwide antimicrobial susceptibility surveillance for
Neisseria gonorrhoeae in Brazil, 2015-16. J Antimicrob Chemother.

[8] Allen VG, Farrell DJ, Rebbapragada A, Tan J, Tijet N, Perusini SJ (2011). Molecular analysis of antimicrobial resistance mechanisms in
Neisseria gonorrhoeae isolates from Ontario, Canada. Antimicrob Agents Chemother.

[9] Ng LK, Martin I, Liu G, Bryden L (2002). Mutation in 23S rRNA associated with macrolide resistance in
Neisseria gonorrhoeae. Antimicrob Agents Chemother.

[10] Barros dos Santos KT, Skaf LB, Justo-da-Silva LH, Medeiros RC, Francisco RDS, Caniné MCA (2019). Evidence for clonally associated increasing rates of azithromycin
resistant Neisseria gonorrhoeae in Rio de Janeiro, Brazil. Biomed Res Int.

