# Supplementary material for: Temporal evolution of antimicrobial resistance among Neisseria gonorrhoeae clinical isolates in the most populated South American Metropolitan Region
Source: Mem Inst Oswaldo Cruz. 2019 Aug 12;114:e190079. doi: 10.1590/0074-02760190079 (PMC6690644; doi:10.1590/0074-02760190079)
Supplement: Supplementary file 1 [file 1678-8060-mioc-114-e190079-s.pdf]

TABLE

Antimicrobial susceptibility profile of *Neisseria gonorrhoeae* clinical isolates included in the study

Antimicrobial agent (n° of isolates tested)	Range (mg/L)	MIC_{50} (mg/L)	MIC_{90} (mg/L)	S ≤ / R >[§] (mg/L)	Category (%)[#]	
					S	R
Penicillin (n = 124)	≤ 0.015 - > 4	4	> 4	0.06/1	10.4	59.6
Ceftriaxone (n = 124)	≤ 0.001 - 0.06	0.002	0.015	0.125/0.125	100.0	0.0
Azitromycin (n = 119*)	≤ 0.015 - > 1	0.25	0.5	0.25/0.5	68.9	6.7
Ciprofloxacin (n = 124)	≤ 0.00025 - > 2	0.002	> 2	0.03/0.06	74.1	15.3

§: clinical breakpoints for *N. gonorrhoeae* according to the European Committee on Antimicrobial Susceptibility Testing (EUCAST, 2015). S: susceptible; R: resistant; #: results were interpreted according to the EUCAST clinical breakpoints, version 5.0 (2015); *: five isolates became unviable during the study.

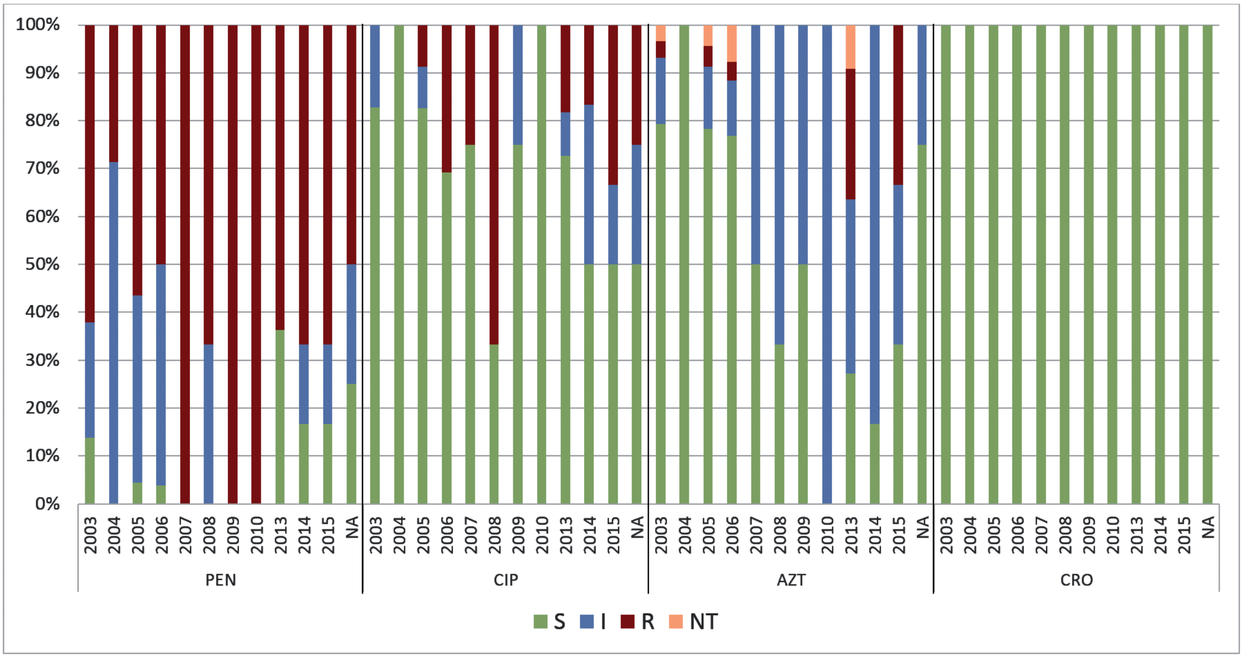

Fig. 1: temporal evolution of antimicrobial susceptibility to penicillin (PEN), ciprofloxacin (CIP), azithromycin (AZT), and ceftriaxone (CRO) among 124 *Neisseria gonorrhoeae* during 12 years. NA: data not available; S: susceptible; I: intermediate; R: resistant; NT: not tested.

CC BY

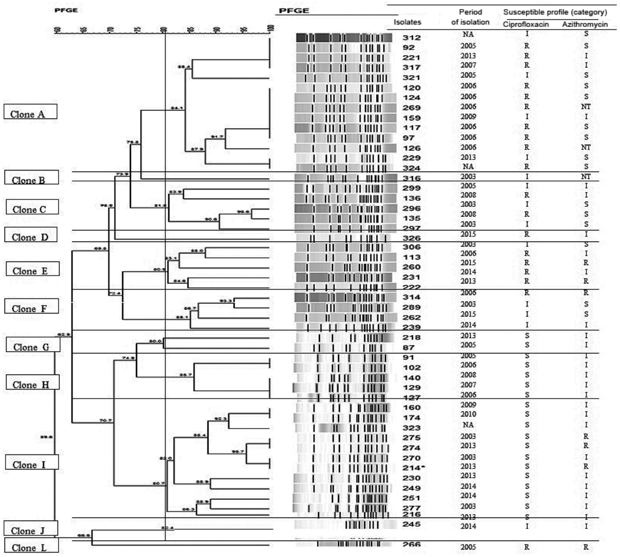

Fig. 2: similarity cluster of ciprofloxacin- and azithromycin-non-susceptible *Neisseria gonorrhoeae* clinical isolates after DNA digestion using SpeI. Horizontal lines indicate different clusters using 80% of similarity. NA: data not available; NT: not tested (isolates became unviable during the study).
